# Plasma-activated Ringer’s Lactate Solution Displays a Selective Cytotoxic Effect on Ovarian Cancer Cells

**DOI:** 10.3390/cancers12020476

**Published:** 2020-02-18

**Authors:** Alina Bisag, Cristiana Bucci, Sara Coluccelli, Giulia Girolimetti, Romolo Laurita, Pierandrea De Iaco, Anna Myriam Perrone, Matteo Gherardi, Lorena Marchio, Anna Maria Porcelli, Vittorio Colombo, Giuseppe Gasparre

**Affiliations:** 1 Department of Industrial Engineering, Alma Mater Studiorum-University of Bologna, 40136 Bologna, Italy; alina.bisag@unibo.it (A.B.); cristiana.bucci@unibo.it (C.B.); sara.coluccelli2@unibo.it (S.C.); matteo.gherardi4@unibo.it (M.G.); vittorio.colombo@unibo.it (V.C.); 2 Centro di Studio e Ricerca sulle Neoplasie Ginecologiche, Alma Mater Studiorum-University of Bologna, 40138 Bologna, Italy; pierandrea.deiaco@unibo.it (P.D.I.); myriam.perrone@aosp.bo.it (A.M.P.); lorena.marchio2@unibo.it (L.M.); annamaria.porcelli@unibo.it (A.M.P.);; 3 Department of Medical and Surgical Sciences, Alma Mater Studiorum-University of Bologna, 40138 Bologna, Italy; 4 Department of Pharmacy and Biotechnology, Alma Mater Studiorum-University of Bologna, 40126 Bologna, Italy; 5 Unit of Gynecologic Oncology, S. Orsola-Malpighi Hospital, 40138 Bologna, Italy; 6 Center for Applied Biomedical Research, Alma Mater Studiorum-University of Bologna, 40138 Bologna, Italy; 7 Interdepartmental Center for Industrial Research Advanced Mechanical Engineering Applications and Materials Technology, Alma Mater Studiorum-University of Bologna, 40136 Bologna, Italy; 8 Interdepartmental Center for Industrial Research Life Sciences and Technologies for Health, Alma Mater Studiorum-University of Bologna, 40064 Ozzano dell’Emilia, Italy; 9 Interdepartmental Center for Industrial Research Agrifood, Alma Mater Studiorum-University of Bologna, 40126 Bologna, Italy

**Keywords:** cold atmospheric pressure plasma, plasma medicine, plasma-activated Ringer’s lactate solution, ovarian cancer, cytotoxicity, selectivity

## Abstract

Epithelial Ovarian Cancer (EOC) is one of the leading causes of cancer-related deaths among women and is characterized by the diffusion of nodules or plaques from the ovary to the peritoneal surfaces. Conventional therapeutic options cannot eradicate the disease and show low efficacy against resistant tumor subclones. The treatment of liquids via cold atmospheric pressure plasma enables the production of plasma-activated liquids (PALs) containing reactive oxygen and nitrogen species (RONS) with selective anticancer activity. Thus, the delivery of RONS to cancer tissues by intraperitoneal washing with PALs might be an innovative strategy for the treatment of EOC. In this work, plasma-activated Ringer’s Lactate solution (PA-RL) was produced by exposing a liquid substrate to a multiwire plasma source. Subsequently, PA-RL dilutions are used for the treatment of EOC, non-cancer and fibroblast cell lines, revealing a selectivity of PA-RL, which induces a significantly higher cytotoxic effect in EOC with respect to non-cancer cells.

## 1. Introduction

Epithelial Ovarian Cancer (EOC) is a relatively rare disease with the highest incidence rate in Western countries such as Europe and North America (8 cases per 100,000) [1]. It is the most lethal and silent gynecological tumor that originates from the epithelium of the ovary, fallopian tubes or the peritoneum [2,3]. About 75% of affected women are diagnosed at advanced stages (III-IV) [3], with a survival rate of 29% within 5 years from diagnosis [4,5]. Furthermore, the spread of cancer to secondary sites is a common complication that contributes to the diffusion of the disease to the peritoneal cavity [6,7]. Standard of care in advanced EOC, since the 1980s, is the combination of surgical cytoreduction followed by first-line platinum-taxane chemotherapy [5,8]. Despite the improvements in survival rates [9], these conventional therapies cannot eradicate the disease [4,8]. However, innovations in the surgical and pharmacological field are creating the conditions to treat this type of neoplastic invasion. This could be accomplished by infusing chemotherapy directly in the peritoneal cavity during surgery, such as in the case of Hyperthermic Intraperitoneal Chemotherapy (HIPEC) [4,9,10,11,12]. This procedure allows one to perform a washing of the abdominal cavity by delivering locally a chemotherapeutic solution [13,14]. Despite the promising results of intraperitoneal chemotherapy administration, the development of efficacious solutions is a cogent issue in order to limit severe drug side effects and overcome chemoresistance.

Plasma-activated liquids (PALs) are produced by electrical discharge in the gas–liquid interface; when high voltage is applied, plasma filaments are generated in the gas phase, leading to the formation of a flow of free radicals, electrons, ions, reactive species and UV radiation. The exposure of a liquid to a plasma induces the production of reactive oxygen and nitrogen species (RONS), like nitrites (NO_2_^−^), nitrates (NO_3_^−^), peroxynitrites (OONO^−^) ozone (O_3_), singlet oxygen (^·^O_2_), hydroxyl radicals (^·^OH) and hydrogen peroxide (H_2_O_2_) [15]. These RONS have been shown to exert a significant role in cancer therapy due to their triggering of cell death mechanisms [16,17]. It was observed in vitro and in vivo that PALs can induce a selective anticancer effect [18,19,20] likely related to the different basal ROS concentration in cancer and non-cancer cells, as the higher metabolic status typical of cancer cells would render them unable to tolerate any increase in oxidative stress, such as the one caused by RONS in PALs [21,22].

PAL treatments turned out to be effective in terms of anti-tumor activity against EOC cells, inhibiting their proliferation and compromising their metastatic potential [20,21,23,24]. In the perspective to propose PALs in clinical applications, it is necessary to select liquids to be exposed to plasma suitable to the clinical phase, such as physiological or Ringer’s Lactate solutions (RL), an intravenous fluid usually used to treat hypovolemia and metabolic acidosis [25]. Tanaka et al. [26] first proposed the use of RL, whose simple composition (NaCl, KCl, CaCl_2_ and lactate) makes it adoptable for the production of PAL, avoiding the possible influence of more organic medium components on its final biological effect [27]. It has been demonstrated that plasma-activated Ringer’s Lactate solutions (PA-RL) exhibit an anti-tumor effect in lung, mammary, ovarian cancer cells as well as in glioblastoma in vitro [25,26,28], and in pancreatic and cervical cancer in vivo [26,29]. Several studies demonstrated that the effects of PA-RL may be ascribed to RONS, together with the activation of lactate [26,28]. All these results suggest that the use of PA-RL may represent a new potential therapeutic strategy for intraperitoneally disseminated cancers. Nonetheless, PA-RL selective cytotoxicity on EOC cells remains to be assessed. Indeed, the capability of an anti-neoplastic drug to act exclusively on cancer cells is essential to preserve the healthy tissue counterpart, [23,30,31], making this aspect one of the most important for the application of PA-RL to EOC treatment [25].

In this study, RL was exposed to plasma generated by a multiwire plasma source used for the first time to produce PA-RL. EOC and non-cancer cells lines were subjected to treatment with PA-RL dilutions in order to evaluate their sensitivity and define a PA-RL selective window. Moreover, we dissected whether PA-RL-induced cell injury may depend on two of the major produced and studied reactive species (H_2_O_2_ and NO_2_^−^), or on the pH change caused by RL. Hence, we further showed that the response of our models to the high oxidative stress caused by PA-RL treatment may be explained analyzing the antioxidant response, which may point to the mechanisms responsible for cancer cells-specific PA-RL toxicity.

## 2. Results

### 2.1. Electrical Characterization of the Multiwire Plasma Source and Chemical Features of PA-RL

**Figure 1:**
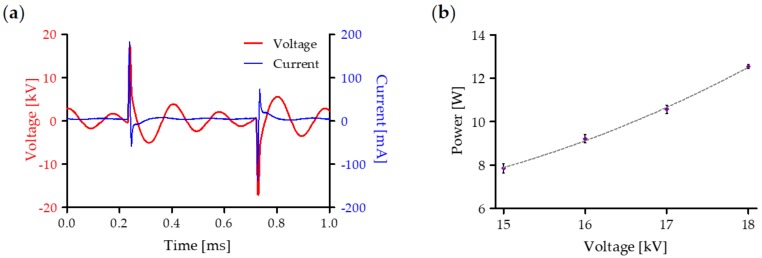
Electrical characterization of plasma source during treatment of Ringer’s Lactate (RL) solution: (**a**) representative voltage (red) and current (blue) waveforms at 18 kV and 1 kHz and (**b**) power values as a function of the applied voltage. Data are presented as mean ± SEM (*n* = 3).

To evaluate the average power of the plasma discharge, the temporal evolution of voltage and current waveforms was recorded during the treatment of RL solution (Figure 1a). Subsequently, data were used for the calculation of the average power as a function of the applied voltage (Figure 1b); the resulting function presents a quadratic behavior according to B. Dong et al. [32].

**Figure 2:**
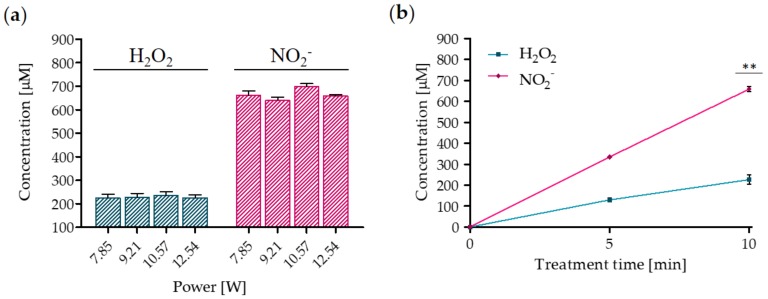
Plasma treatment leads to the formation of H_2_O_2_ and NO_2_^−^. (**a**) Reactive oxygen and nitrogen species (RONS) concentration as a function of the average power after 10 min of plasma treatment. Data are presented as mean ± SEM (*n* = 3). (**b**) H_2_O_2_ and NO_2_^−^ concentrations as a function of treatment time. Data are presented as mean ± SEM (*n* = 3) and statistical significance is specified with asterisks (** *p* ≤ 0.001 as determined by a paired Student’s *t*-test, versus the 5 min treatment).

RONS variation induced by plasma treatment for different average power values is shown in Figure 2a: the concentration of both H_2_O_2_ and NO_2_^−^ measured in the liquid phase resulted in not being affected by the average power in the range of 7.85-12.54 W. Conversely, they strongly depended on the treatment time (Figure 2b). More specifically, the H_2_O_2_ and NO_2_^−^ concentrations increased linearly with the treatment time and reached a maximum of 226 ± 12.46 μM and 659 ± 15.19 μM, respectively. Furthermore, the ratio NO_2_^−^/H_2_O_2_ was 2.91 in the liquid treated for 10 min.

**Figure 3:**
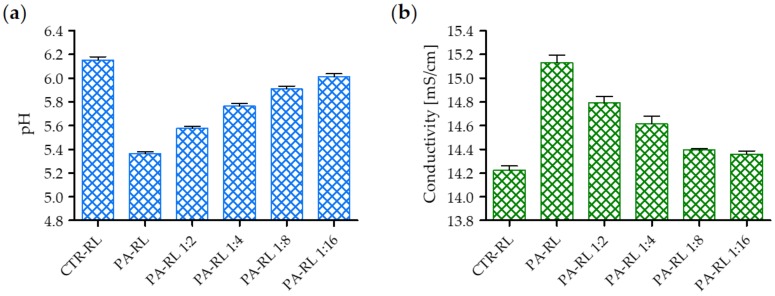
Chemical characterization of plasma-activated RL (PA-RL) and its dilutions after 10 min of plasma treatment at 18 kV. (**a**) pH and (**b**) conductivity as a function of serial dilutions. Data are presented as mean ± SEM (*n* = 3).

In addition, pH and conductivity of the PA-RL and its dilutions are reported in Figure 3. After 10 min of plasma treatment, PA-RL pH decreased to 5.36 (PA-RL) (Figure 3a), whereby only dilutions starting from 1:4 were used for subsequent cell treatments. Plasma also induced an increase of conductivity up to 15.13 mS/cm (Figure 3b).

### 2.2. Evaluation of Plasma Discharge Behavior and Emission by Мeans of Low-Speed and High-Speed Filter Imaging

**Figure 4:**
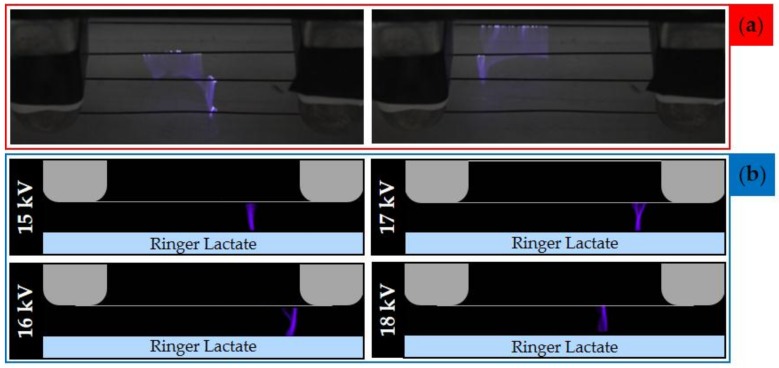
Low-speed images and high-speed (HS) filter images of the multiwire plasma discharge during RL treatment. (**a**) Picture of plasma generated during the treatment of PA-RL with an applied voltage of 18 kV and 30 fps. (**b**) HS filter images of plasma filaments for different values of applied voltage (between 15 to 18 kV) and 100 fps.

Low-speed imaging was performed to assess the global behavior of plasma filaments generated during the treatment. Plasma discharge consisted of random streamers generated between the wire-electrodes and impinging on the liquid surface (Figure 4a). To further investigate the plasma discharge, a high-speed camera equipped with a 402 nm filter was used to visualize the emission of plasma in contact with the RL during treatment. The filter wavelength was selected to highlight specifically the emission of vibrationally excited nitrogen molecules, precursors of reactive nitrogen species generated in the liquid phase. In Figure 4b representative HS filter images of the multiwire discharge generated applying different voltages are shown. In all investigated cases, it is possible to observe that single filaments were randomly generated between the high voltage wire electrode and the liquid surface. Moreover, no relevant differences could be observed upon varying the input voltage between 15 and 18 kV.

### 2.3. PA-RL Displays a Cytotoxic Effect on EOC Cell Lines, which Does not Depend Exclusively on Hydrogen Peroxide or Nitrites

We first tested three different PA-RL dilutions (1:4, 1:8 and 1:16) on two different EOC cell lines, namely OV-90 and SKOV-3, over time, with the aim to understand if PA-RL exerted a cytotoxic effect and if this was dependent on the dilution, i.e., on the concentration of reactive species to which cells were exposed. After two hours of exposure to PA-RL, both OV-90 and SKOV-3 showed a decrease in viability only when the 1:4 dilution was used, whereas only OV-90 appeared to respond early to PA-RL even at higher dilutions. Both OV-90 and SKOV-3 cells were observed to be similarly affected in terms of viability when treated with the three PA-RL dilutions after 72 h of exposure, displaying a dose-dependent response that was more evident in the OV-90 cell line, and showing a dramatic decrease in viability, which was between 80% and 95% in the two cell lines, even with the more diluted PA-RL (1:16, Figure 5a). Overall, SKOV-3 cells only initially appeared to be less sensitive to the treatment, as their viability decreased in time in a more delayed fashion, unlike OV-90, but at 72 h both cell populations showed to be severely affected by PA-RL. The viability of both EOC cell lines at 24 and 48 h after treatment is shown in Supplementary Figure S1a to highlight the time-dependent effect.

**Figure 5:**
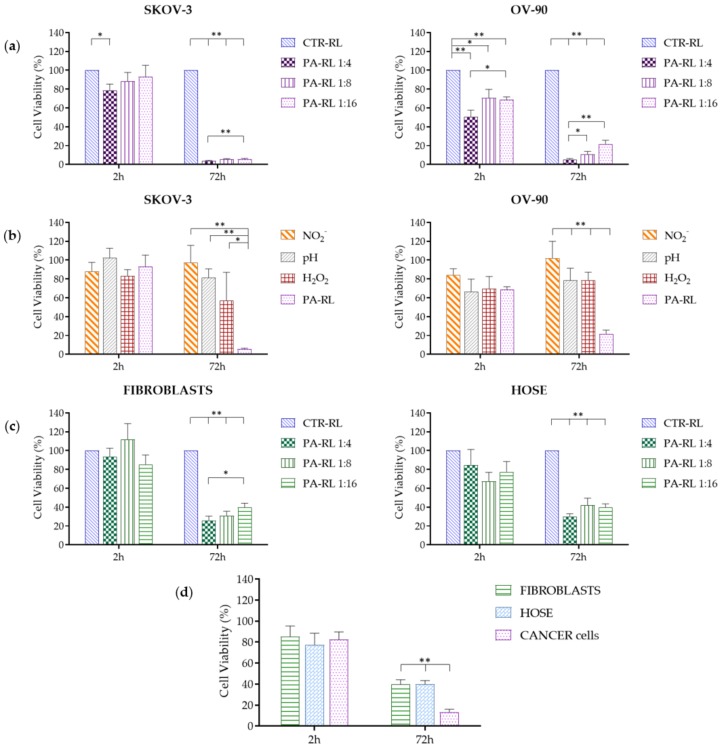
PA-RL displays a selective cytotoxic effect on Epithelial Ovarian Cancer (EOC) cell lines. (**a**) Viability of SKOV-3 (*n* = 7) and OV-90 (*n* = 9) cell lines treated with PA-RL dilutions (1:4, 1:8 and 1:16). Data are mean ± SEM normalized on the corresponding control in RL (CTR-RL). (**b**) Viability of SKOV-3 and OV-90 cell lines treated with PA-RL 1:16 and synthetic solutions at dilution 1:16. H_2_O_2_-supplemented RL, NO_2_^−^-supplemented RL and pH-adjusted RL solutions were diluted in RL to obtain the final treatment solutions. Data are mean ± SEM (*n* = 3) normalized on the corresponding CTR-RL. (**c**) Viability of non-cancer cells, namely human fibroblasts (*n* = 9) and HOSE (*n* = 4) treated with different PA-RL dilutions (1:4, 1:8 and 1:16). Data are mean ± SEM normalized on the corresponding CTR-RL. (**d**) PA-RL 1:16 efficacy on cell viability in non-cancer and EOC cell lines. Cell viability was normalized to the CTR-RL at 2 h and plotted as percentage relative to the corresponding CTR-RL, for both time points. In each panel, data are mean ± SEM and statistical significance is specified with asterisks (* *p* ≤ 0.05, ** *p* ≤ 0.001 as determined by a paired Student’s *t*-test).

We hence decided to verify whether the cytotoxic effects of PA-RL could be ascribed mainly to either of the two components we could easily compare PA-RL with, namely hydrogen peroxide and nitrites. The scope of this analysis was to understand whether the complexity of PA-RL might be substituted by a simpler solution of one of the two components, such as for instance H_2_O_2_, more readily available in hospital settings. We also verified whether the observed toxicity may be due to pH change: we hence obtained RL solutions containing hydrogen peroxide and nitrites at the same concentrations as measured in the PA-RL 1:16 dilution, as the latter was shown to have a toxic effect on cancer cells. A RL solution of the same pH of the 1:16 dilution was prepared to which both OV-90 and SKOV-3 were exposed for 2 h, then cultured for the subsequent 72 h. In these conditions, OV-90 cells were confirmed to undergo a more immediate decrease in viability, consistent among the different treatments, of about 20%–30% after 2 h exposure, whereas SKOV-3 cells appeared to suffer only from a mild to no loss of viability during the same time frame (Figure 5b). After 72 h, nitrites were not shown to have any effect on cell viability for both cancer cell lines, whereas pH and H_2_O_2_ only mildly inhibited growth with respect to both nitrites and control. We overall validated that only PA-RL was able to dramatically reduce viability of both cancer cell lines (Figure 5b), suggesting the different RONS therein contained may have a synergistic effect in the induction of cytotoxicity, and that the complexity of the PA-RL may not be substituted by the synthetic solutions we here utilized.

### 2.4. PA-RL Is Selective for EOC Cells

In order for PA-RL to find application in the clinics, one of the main requisites to be fulfilled is that its cytotoxic action ought to be specific for cancer cells, while sparing non-cancer cells, particularly those of the connective tissues, so to allow recovery of the wounds within the pelvic cavity. We hence next questioned whether our PA-RL may display such a specific effect, and attempted to prove so by using two different cell models, namely the non-cancer epithelial cell lines of ovarian origin (HOSE), as the counterpart for both EOC models, and two different human immortalized fibroblast lines, to gauge the response to PA-RL of the tissue mesenchymal component. Therefore, we tested the same three different PA-RL dilutions at the same time points as the EOC cell lines; surprisingly, we observed a similar rate of decrease of both fibroblasts and HOSE cells with respect to their viability, which was evident as a late response (i.e., not observed at the 2 h exposure), in a dose-independent fashion at 72 h, ranging between 60% and 70%, with the highest survival at the 1:16 dilution (Figure 5c, Figure S1b). The 1:16 dilution, therefore, was deemed as the best compromise to obtain a high degree of mortality in cancer cells while sparing both the non-cancer epithelial population and fibroblasts. Indeed, when we compared the decrease in cell viability of cancer versus non-cancer cells, the effects of PA-RL were shown to be significantly different (Figure 5d, Figure S1c).

### 2.5. Differentially Activated Antioxidant Defenses Mechanisms May Underlie Cancer Cells-Specific PA-RL Toxicity

Last, we attempted to understand the mechanisms underlying the different responses in terms of the viability of cancer with respect to non-cancer cells. It is widely accepted that cancer cells withstand a higher degree of oxidative stress during their fast proliferation, for which they have been shown in several contexts to display higher levels of antioxidant proteins. The latter should act as a defense mechanism against the excess of radical species, since overloading neoplastic cells with radicals may lead to an oxidation-mediated collapse [33].

**Figure 6:**
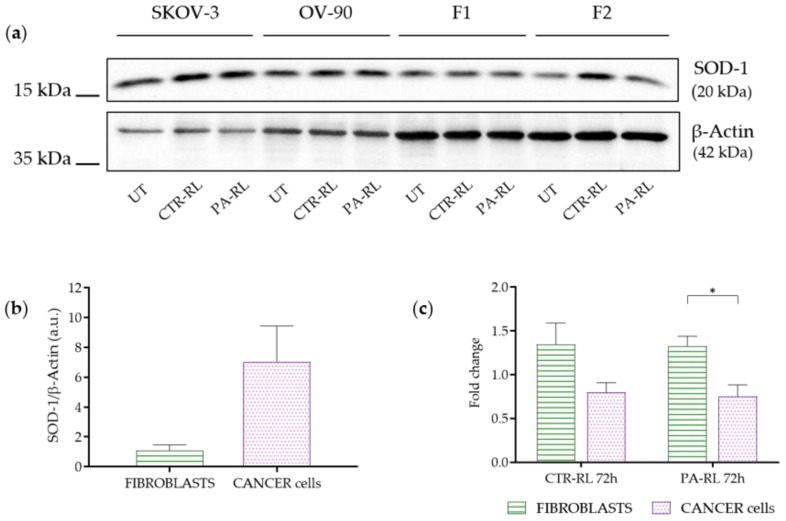
PA-RL solution induces an increase in Superoxide Dismutase-1 (SOD-1) expression in fibroblasts but not in EOC cell lines. (**a**) Western blot analysis of EOC cell lines and fibroblasts (F1 and F2) at 72 h after treatment with PA-RL 1:16 (UT, untreated cells). A representative experiment of three is shown. (**b**) SOD-1 levels in untreated fibroblasts and cancer cell lines. Histograms show densitometric values of the SOD-1 protein normalized to the β-actin used as a loading control. All data are presented as mean ± SEM of three independent experiments. (**c**) Relative densities of SOD-1 and β-actin were measured using densitometric analysis. SOD-1 levels of CTR-RT and PA-RL 1:16 after 72 h of treatment were normalized to β-actin and plotted as fold change relative to the untreated (UT) sample. All data are presented as mean ± SEM of three independent experiments. Statistical significance is specified with asterisks (* *p* ≤ 0.05 as determined by a paired Student’s *t*-test).

We hence measured the levels of one of the most active cytosolic antioxidant enzymes involved in radical species detoxification, namely superoxide dismutase-1 (SOD-1), both in the two EOC cell lines and in human fibroblasts, to ascertain if indeed cancer cells expressed higher levels of the protein (Figure 6a, Figure S2). As expected, we observed increased levels of SOD-1 in cancer cells versus human fibroblasts, although this did not reach statistical significance in our Western blot analysis (Figure 6b, Figure S1d). We then proceeded to treat all four cell lines with PA-RL, or with RL alone, and observed the changes in SOD-1 levels at the 72 h time point. Indeed, a statistically significant increase in SOD-1 expression was evident in fibroblasts only, when these cells were treated with PA-RL, whereas the levels of the enzyme remained unchanged in cancer cells (Figure 6c, Figure S1e), suggesting that here the antioxidant response has likely reached a plateau over which enzymes levels may not be increased. Fibroblasts, instead, may be able to adapt to the oxidative burst by increasing SOD-1 levels, albeit proving this unequivocally warrants further functional experiments. It must be noted that, although not statistically significant, a trend for an increase in SOD-1 levels in fibroblasts but not in cancer cells may be observed in response to RL treatment alone, with a similar fold increase as with PA-RL (Figure 6c, Figure S1e).

On one hand, this may indicate that RL treatment may be synergistic with PALs to trigger an antioxidant response; on the other, it is of modest relevance what is the causative hit to induce such an enzymatic increase, since the activated mechanism would still be protective against RONS.

## 3. Discussion

In this work, we produced a PA-RL through a multiwire plasma source, whose main innovative feature is its ability to work without the use of a technical gas, while allowing to treat 20 mL of liquid [28,34,35,36]. Moreover, the source architecture we here propose can be easily scaled in order to produce volumes of activated solution higher than 20 mL. The interaction of plasma discharges with liquid substrates leads to the formation of a high concentration of RONS [37,38]; these latter are formed in chemical reactions involving species generated in the plasma (gas phase) and diffusing into the liquid. As an example, the formation of NO_2_^−^ involves the gas phase reaction of NO with OH molecules, resulting in the production of HNO_2_ that dissolves in the liquid phase and leads to the formation of NO_2_^−^. While atmospheric NO is generally produced via the Zeldovich mechanism and requires high temperatures (1300 °C), the same process in non-equilibrium plasma can take place close to room temperature due to the production of a high number of vibrationally excited N_2_ molecules [38]; the presence of these molecules favors the breakage of N-N bonds to release N atoms that react with O_2_ and ·O to produce NO. As shown by M. Simek et al. in the case of a plasma discharge working in environmental air, vibrationally excited N_2_ molecules emit light at a wavelength around 400 nm (second positive system, C^3^Π_u_→B^3^Π_g_) [39]. In this respect, Figure 4b confirms the presence of vibrationally excited N_2_ molecules and thus the gas phase origin of the NO_2_^−^ measured in the PAL. The plasma treatment of RL induced the production of RONS and a decrease of pH, and PA-RL was tested on both cancer and non-cancer cells in vitro to validate a cytotoxic effect specific for EOC cells.

The issue of selectivity in the search for anticancer therapies has always been a cogent one, and a plethora of research lines have focused on detecting the molecular differences between normal and transformed cells on which to design a targeted approach. One such difference has been shown to be the capacity of cancer cells to withstand the oxidative stress they come to face due to their metabolic rewiring, their high proliferation rate, and to the microenvironment conditions that quickly build up around a progressing tumor mass [33,40]. Albeit our data are preliminary in terms of understanding the molecular causes for a relevant degree of selectivity of PA-RL, they point to a different ability of non-cancer cells to regulate the enzymatic milieu responsible for reactive species detoxification, unlike in neoplastic cells. This may reveal the triggering of a salvage mechanism when a boost of RONS is provided from external sources. Of note, we did wonder whether hydrogen peroxide alone, or nitrites, may have the same effect as PA-RL, but we showed this not to be the case, pointing to the need for a complex source of RONS to achieve the steep decrease in viability we observed in our cell models.

Whatever the cause, which warrants further investigation, we believe our most relevant data are those showing a consistently higher effect of the PA-RL we generated and characterized on the two OC cells lines compared to both non-cancer ovarian cells and fibroblasts. In this regard, the ability of PAL to suppress ovarian cancer metastases when injected intraperitoneally in a mouse model was previously reported [24]. Yet, no evidence is available on the safety of PAL intraperitoneal administration in humans [41], supporting the need to shift the main focus of the plasma onco-medicine on liquids applicable to the clinical practice. This holds particularly true in OC, where spreading of the advanced stage disease within the pelvic cavity of the patient is often the case through the occurrence of micro-lesions [42], as washing the cavity with PA-RL may significantly reduce tumor burden, while sparing the non-cancer component.

## 4. Materials and Methods

### 4.1. Plasma Device and Electrical Characterization

**Figure 7:**
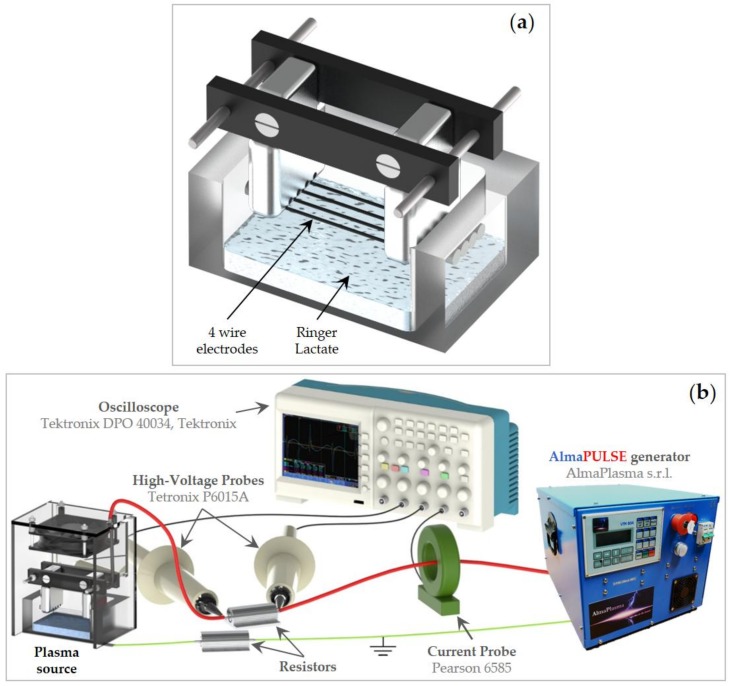
(**a**) Illustration of the high voltage electrodes and RL and (**b**) layout of the setup used for electrical characterization.

PA-RL was produced by exposing RL (Fresenius Kabi Italia S.r.l.) to a micropulsed plasma discharge (Figure 7a). The high voltage electrodes consist of four steel wires individually fixed on aluminum supports and connected to high voltage generator through a ballast resistor of 70 kΩ; while the ground electrode consists of an aluminum sheet fixed on the bottom of the 5 mm thickness vessel containing the liquid substrate and is connected to the ground through a resistor of 30 kΩ. A polymethylmethacrylate (PMMA) box encased the plasma source to guarantee a controlled atmosphere during treatment; moreover, the box was equipped with a fan.

The setup reported in Figure 7b was used to measure the time evolution of the plasma discharge electrical parameters using a 5 mm gap value between the high voltage electrodes and the liquid surface. The plasma device was driven by a micropulsed high voltage generator (AlmaPULSE, AlmaPlasma s.r.l., Bologna, Italy) delivering a peak voltage of 18 kV, pulse duration FWHM (Full Width at Half Maximum) of 8 μs and pulse repetition rate set at 1 kHz. In addition, a high voltage probe (Tektronix P6015A) was used to measure the voltage, while the discharge current was measured by the means of a current probe (Pearson 6585). Both probes were connected to an oscilloscope (Tektronix DPO 40034). The average power (P) over a period (T) was calculated starting from current (I) and voltage (V) measurements:

(1) $$P=\frac{1}{T}\int_{T}^{\;}VI\;dt$$

### 4.2. PA-RL and Synthetic Solutions Production

20 mL of RL were exposed to plasma for 10 min using a 5 mm gap between the high voltage wire electrodes and the liquid surface to produce PA-RL. The pulse repetition frequency (PRF) was fixed at 1 kHz, while the peak voltage (PV) was set at 18 kV with fan always on. After plasma treatment, quantitative measurements of H_2_O_2_ and NO_2_^−^ were performed using Amplex^®^ Red Hydrogen Peroxide Assay Kit (Thermo Fisher Scientific #A22188, Waltham, MA, USA) and Nitrite/Nitrate colorimetric assay (ROCHE #11746081001, Basel, Switzerland) [43], respectively. In addition, before and after RL exposure to plasma, pH and conductivity were evaluated by the means of inoLab^®^ pH 7110 and Oakton Instrument: Con 6+ Meter, respectively. Subsequently, PA-RL was diluted by preparing two-fold serial dilutions (1:4, 1:8 and 1:16) in RL and their effect was tested on our cell models.

Synthetic solutions were also prepared; two different RL solutions were supplemented with 226 μM of H_2_O_2_ (Sigma-Aldrich, #216763, St. Louis, MO, USA) and 659 μM of NO_2_^−^ (Alfa Aesar by Thermo Fisher (Kandel) GmbH, #43015-, Karlsruhe, Germany), the same concentrations generated by plasma treatment in PA-RL. An additional synthetic solution was prepared by adjusting the pH of RL to 5.36 with a solution of 0.01 M HCl, according to the pH-value gauged in PA-RL. The above mixtures were diluted in RL as mentioned before, thereafter EOC cell lines were treated with the synthetic solutions at dilution 1:16.

### 4.3. Low-Speed and High-Speed Filter Imaging

**Figure 8:**
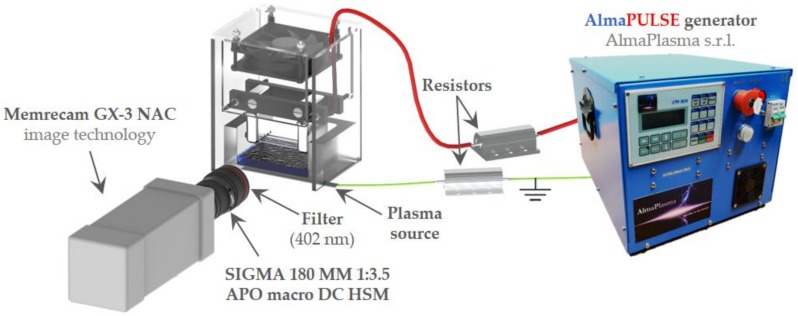
High-speed filter imaging setup.

A low-speed camera (Nikon D800, Shinjuku, Tokyo, Japan) was operated at 30 fps for the evaluation of the behavior of the plasma discharge, as reported in Figure 4a. The high-speed filter imaging setup, employed for the characterization of plasma source (Figure 8), was composed of a high-speed (HS) camera (Memrecam GX-3 NAC image technology) operated at 100 fps and 1/200 shutter time. Additionally, a camera lens (SIGMA 180 MM 1:3.5 APO macro DC HSM) was used and a 402 nm filter (CHROMA ET402/15x, #327585, Bellows Falls, VT, USA) was positioned in front of the latter to evaluate the emission of N_2_(C^3^Π_u_→B^3^Π_g_) second positive system near 400 nm. During HS-filter imaging, the focus of the acquisitions was set in correspondence of the electrode closer to the filter.

### 4.4. Cell Lines and Culture Conditions

Human EOC cell lines SKOV-3 and OV-90 were purchased from ATCC^®^ (Manassas, VA, USA). The HOSE cell line was purchased from ScienCell Research Laboratories, Inc. (Carlsbad, CA, USA) and two lines of immortalized fibroblasts (F1 and F2) derived from two patients skin biopsies, obtained within the context of a study approved by the Independent Ethics Committee of the S. Orsola Hospital (107/2011/U/Tess) were used as non-cancer controls.

EOC cell lines, HOSE and fibroblasts were grown respectively in Roswell Park Memorial Institute 1640 medium (RPMI, EuroClone, Milan, Italy), Ovarian Epithelial Cell Medium (OEpiCM, ScienCell Research Laboratories, Inc., Carlsbad, CA, USA) and Dulbecco’s modified Eagle’s medium (DMEM High glucose, EuroClone). They were all supplemented with 10% heat-inactivated fetal bovine serum (FBS), 2 mM L-glutamine, 100 U/mL penicillin and 100 µg/mL streptomycin (EuroClone). Cells were maintained in an incubator with a humidified atmosphere of 5% CO_2_ at 37 °C.

### 4.5. Cell Treatment and Viability Assay

SKOV-3 (2 × 10^3^ cells/well), OV-90 (4 × 10^3^ cells/well), HOSE (7 × 10^3^ cells/well), F1 (9 × 10^3^ cells/well) and F2 (1 × 10^4^ cells/well) were seeded in 96-well plates in complete medium. After 24 h, cells were treated with 100 μL of freshly produced PA-RL at different dilutions (1:4, 1:8 and 1:16) and RL. After 2 h of treatment, cells were washed with phosphate buffered solution (PBS) and cultured in complete medium at 37 °C and 5% CO_2_. Cell viability was assessed after the exposure of cells to treatments and measured by using Sulforhodamine B (SRB; Sigma-Aldrich, #S1402, St. Louis, MO, USA) assay at 2, 24, 48 and 72 h after treatment. Treated cells were fixed with 50% cold trichloroacetic acid (TCA) for 1 h, washed 5 times with distilled water to eliminate TCA, and stained with 0.4% SRB for 30 min. Protein-bound dye was dissolved in 10 mM pH 10.5 Tris base solution after four washes with 1% acetic acid to remove unbound dye. SRB was used to determine cell density, based on the measurement of cell protein content. Absorbance values were determined at 570 nm using a 96-well Multilabel Plate Reader VICTOR^3^ (1420 Multilabel Counter-PerkinElmer, Turku, Finland). The percentage of viability was calculated considering RL-treated cells as the control (CTR-RL).

### 4.6. SDS-PAGE and Western Blot Analysis

Cells were seeded and after 24 h treated with RL solution and freshly produced PA-RL 1:16 dilution. At this point, an untreated (UT) sample was collected for each cell lines. Two hours after treatments, cells were washed in PBS and cultured in their own complete medium at 37 °C and 5% CO_2_. After 72 h, CTR-RL and PA-RL treated samples of each cell line were collected. Total lysate was obtained by using RIPA buffer (50 mM Tris–HCl pH 7.4, 150 mM NaCl, 1% SDS, 1% Triton X-100 and 1 mM EDTA pH 7.6) supplemented with protease inhibitors (ThermoFisher #A32955, Waltham, MA, USA). The protein concentration was determined by a Lowry protein assay (Bio-Rad #5000116, Hercules, CA, USA). Proteins (30 μg) were separated by using SDS-PAGE on a 12% polyacrylamide gel and then transferred onto a Trans-Blot Turbo Midi Nitrocellulose membrane (Bio-Rad #1704159). Membranes were blocked with 5% TBS-Tween/milk (0.1% Tween 20 (Sigma-Aldrich #P9416, St. Louis, MO, USA) in Tris Buffered Saline and incubated with the anti-SOD-1 1:1000 (Santa Cruz Biotechnology #sc-11407, Dallas, TX, USA) overnight at 4 °C and subsequently with anti-β-actin 1:10000 (Sigma-Aldrich #A5316) for 1 h at room temperature (RT)). Membranes were washed four times for 5 min using TBS-Tween and then incubated with secondary antibodies (Jackson ImmunoResearch Laboratories #111035144 and #111035146, West Grove, PA, USA), diluted 1:20000 (anti-rabbit) and 1:10000 (anti-mouse) in TBS-Tween for 30 min at RT. Development was performed by using Clarity Western ECL Substrate (Bio-Rad #1705061) and exposing with ChemiDoc XRS+ (Bio-Rad). Protein levels were determined by densitometry of each specific band normalizing on β-actin signal by using ImageJ software (Version 1.5Oi, Bethesda, MD, USA).

### 4.7. Statistical Analyses

Statistical analyses were performed using a Student’s *t*-test. The results were expressed as the mean ± standard error of the mean (SEM; *n* ≥ 3).

## 5. Conclusions

New therapeutic approaches for the treatment of EOC involve the combination of multiple therapies (chemotherapy, antiangiogenic agents and PARP inhibitors). Ovarian cancer cells, however, thrive as they develop resistance against current drugs, through mechanisms that are currently unclear, hence decreasing the long-term efficacy of therapies. Treatment of liquids by means of cold atmospheric pressure plasma, due to their content of RONS, may respond to the requirement for new types of active treatments against ovarian cancer, which may be used in combination with other standard therapies. In this context, the novelty of our approach lies in the use of a well-known clinically suitable fluid, namely RL. We reported that PA-RL produced by exposing RL to plasma has a degree of selectivity for cancer cells compared to fibroblasts, although further investigations need to confirm the exact mechanism underlying such preferential activity.

In conclusion, albeit far from clinical practice, PA-RL may represent a good candidate to respond to the requirement for novel therapies with a local administration, which act on cancer cells with reduced damage on the surrounding healthy tissues.

## Supplementary Materials

The following are available online at https://www.mdpi.com/2072-6694/12/2/476/s1, Figure S1: Viability of (a) SKOV-3 (*n* = 7) and OV-90 (*n* = 9) and (b) fibroblasts (*n* = 9) and HOSE (*n* = 4) cells treated with PA-RL dilutions (1:4, 1:8 and 1:16). Data are mean ± SEM normalized on its respective control in RL (CTR-RL). (c) PA-RL 1:16 efficacy on cell viability in SKOV-3, OV-90, fibroblasts and HOSE cells at 2, 24, 48 and 72 h after treatment. Cell viability was normalized to the CTR-RL at 2 h and plotted as percentage relative to corresponding CTR-RL, for both time points. Data are mean ± SEM. (d) SOD-1 levels in untreated fibroblasts, SKOV-3 and OV-90 cell lines. Histograms represent densitometric values of SOD-1 protein normalized to the β-Actin used as loading control. All data are presented as the mean ± SEM of three independent experiments. (e). Relative densities of SOD-1 and β-Actin were measured using densitometric analysis of the western blots. SOD-1 levels of CTR-RT and PA-RL 1.16 after 72 h of treatment were normalized to β-Actin and plotted as fold change relative to the untreated (UT) sample. All data are presented as the mean ± SEM of three independent experiments (*n* = 3). Statistical significance is specified with asterisks (* *p* ≤ 0.05, ** *p* ≤ 0.001 as determined by paired Student *t*-test), Figure S2: Additional information. Uncropped blot showing the different bands with molecular weight markers for SOD-1 and β-Actin represented in Figure 6a. The image was acquired using ChemiDoc (Bio-Rad). The chemiluminescent blot image was merged with a colorimetric image representing the marker of the same Western blot membrane using Image Lab Software (Bio-Rad).

## References

[1] Reid B.R., Permuth J.B., Sellers T.A. (2017). Epidemiology of ovarian cancer: A review. Cancer Biol. Med..

[2] Van Baal J.O.A.M., Van Noorden C.J.F., Nieuwland R., Van De Vijver K.K., Sturk A., Van Driel W.J., Kenter G.G., Lok C.A.R. (2018). Development of Peritoneal Carcinomatosis in Epithelial Ovarian Cancer: A Review. J. Histochem. Cytochem..

[3] Prat J. (2015). FIGO’s staging classification for cancer of the ovary, fallopian tube, and peritoneum: Abridged republication. J. Gynecol. Oncol..

[4] Jewell A., McMahon M., Khabele D. (2018). Heated Intraperitoneal Chemotherapy in the Management of Advanced Ovarian Cancer. Cancers.

[5] Lheureux S., Gourley C., Vergote I., Oza A.M. (2019). Epithelial ovarian cancer. Lancet.

[6] Sant M., Minicozzi P., Mounier M., Anderson L.A., Brenner H., Holleczek B., Marcos-Gragera R., Maynadié M., Monnereau A., Osca-Gelis G. (2014). Survival for haematological malignancies in Europe between 1997 and 2008 by region and age: Results of EUROCARE-5, a population-based study. Lancet Oncol..

[7] Puiffe M.L., Le Page C., Filali-Mouhim A., Zietarska M., Ouellet V., Tonin P.N., Chevrette M., Provencher D.M., Mes-Masson A.M. (2007). Characterization of ovarian cancer ascites on cell invasion, proliferation, spheroid formation, and gene expression in an in vitro model of epithelial ovarian cancer. Neoplasia.

[8] Rynne-Vidal A., Au-Yeung C.L., Jiménez-Heffernan J.A., Pérez-Lozano M.L., Cremades-Jimeno L., Bárcena C., Cristóbal-García I., Fernández-Chacón C., Yeung T.L., Mok S.C. (2017). Mesothelial-to-mesenchymal transition as a possible therapeutic target in peritoneal metastasis of ovarian cancer. J. Pathol..

[9] Della Pepa C., Tonini G., Pisano C., Di Napoli M., Cecere S.C., Tambaro R., Facchini G., Pignata S. (2015). Ovarian cancer standard of care: Are there real alternatives?. Chin. J. Cancer.

[10] Van Driel W.J., Koole S.N., Sikorska K., Schagen van Leeuwen J.H., Schreuder H.W.R., Hermans R.H.M., de Hingh I.H.J.T., van der Velden J., Arts H.J., Massuger L.F.A.G. (2018). Hyperthermic Intraperitoneal Chemotherapy in Ovarian Cancer. N. Engl. J. Med..

[11] Wu Q., Wu Q., Xu J., Cheng X., Wang X., Lu W., Li X. (2019). Efficacy of hyperthermic intraperitoneal chemotherapy in patients with epithelial ovarian cancer: A meta-analysis. Int. J. Hyperth..

[12] Di Giorgio A., De Iaco P., De Simone M., Garofalo A., Scambia G., Pinna A.D., Verdecchia G.M., Ansaloni L., Macrì A., Cappellini P. (2017). Cytoreduction (Peritonectomy Procedures) Combined with Hyperthermic Intraperitoneal Chemotherapy (HIPEC) in Advanced Ovarian Cancer: Retrospective Italian Multicenter Observational Study of 511 Cases. Ann. Surg. Oncol..

[13] Chang Y.H., Li W.H., Chang Y., Peng C.W., Cheng C.H., Chang W.P., Chuang C.M. (2016). Front-line intraperitoneal versus intravenous chemotherapy in stage III-IV epithelial ovarian, tubal, and peritoneal cancer with minimal residual disease: A competing risk analysis. BMC Cancer.

[14] Tewari D., Java J.J., Salani R., Armstrong D.K., Markman M., Herzog T., Monk B.J., Chan J.K. (2015). Long-Term Survival Advantage and Prognostic Factors Associated with Intraperitoneal Chemotherapy Treatment in Advanced Ovarian Cancer: A Gynecologic Oncology Group Study. J. Clin. Oncol..

[15] Locke B.R., Lukes P., Brisset J.L., Parvulescu V.I., Magureanu M., Lukes P. (2012). Elementary chemical and physical phenomena in electrical discharge plasma in gas-liquid environments and in liquids. Plasma Chemistry and Catalysis in Gases and Liquids.

[16] Graves D.B. (2012). The emerging role of reactive oxygen and nitrogen species in redox biology and some implications for plasma applications to medicine and biology. J. Phys. D Appl. Phys..

[17] Graves D.B. (2014). Reactive species from cold atmospheric plasma: Implications for cancer therapy. Plasma Process. Polym..

[18] Di Meo S., Reed T.T., Venditti P., Victor V.M. (2016). Role of ROS and RNS Sources in Physiological and Pathological Conditions. Oxid. Med. Cell. Longev..

[19] Kaushik N.K., Ghimire B., Li Y., Adhikari M., Veerana M., Kaushik N., Jha N., Adhikari B., Lee S.J., Masur K. (2018). Biological and medical applications of plasma-activated media, water and solutions. Biol. Chem..

[20] Utsumi F., Kajiyama H., Nakamura K., Tanaka H., Mizuno M., Ishikawa K., Kondo H., Kano H., Hori M., Kikkawa F. (2013). Effect of Indirect Nonequilibrium Atmospheric Pressure Plasma on Anti-Proliferative Activity against Chronic Chemo-Resistant Ovarian Cancer Cells In Vitro and In Vivo. PLoS ONE.

[21] Utsumi F., Kajiyama H., Nakamura K., Tanaka H., Mizuno M., Toyokuni S., Hori M., Kikkawa F. (2016). Variable susceptibility of ovarian cancer cells to non-thermal plasma-activated medium. Oncol. Rep..

[22] Laroussi M. (2019). Effects of PAM on select normal and cancerous epithelial cells. Plasma Res. Express.

[23] Utsumi F., Kajiyama H., Nakamura K., Tanaka H., Hori M., Kikkawa F. (2014). Selective cytotoxicity of indirect nonequilibrium atmospheric pressure plasma against ovarian clear-cell carcinoma. Springerplus.

[24] Nakamura K., Peng Y., Utsumi F., Tanaka H., Mizuno M., Toyokuni S., Hori M., Kikkawa F., Kajiyama H. (2017). Novel Intraperitoneal Treatment with Non-Thermal Plasma-Activated Medium Inhibits Metastatic Potential of Ovarian Cancer Cells. Sci. Rep..

[25] Matsuzaki T., Kano A., Kamiya T., Hara H., Adachi T. (2018). Enhanced ability of plasma-activated lactated Ringer’s solution to induce A549 cell injury. Arch. Biochem. Biophys..

[26] Tanaka H., Nakamura K., Mizuno M., Ishikawa K., Takeda K., Kajiyama H., Utsumi F., Kikkawa F., Hori M. (2016). Non-thermal atmospheric pressure plasma activates lactate in Ringer’s solution for anti-tumor effects. Sci. Rep..

[27] Biscop E., Lin A., Van Boxem W., Van Loenhout J., Backer J., Deben C., Dewilde S., Smits E., Bogaerts A. (2019). Influence of Cell Type and Culture Medium on Determining Cancer Selectivity of Cold Atmospheric Plasma Treatment. Cancers.

[28] Tanaka H., Mizuno M., Katsumata Y., Ishikawa K., Kondo H., Hashizume H., Okazaki Y., Toyokuni S., Nakamura K., Yoshikawa N. (2019). Oxidative stress-dependent and -independent death of glioblastoma cells induced by non-thermal plasma-exposed solutions. Sci. Rep..

[29] Sato Y., Yamada S., Takeda S., Hattori N., Nakamura K., Tanaka H., Mizuno M., Hori M., Kodera Y. (2018). Effect of Plasma-Activated Lactated Ringer’s Solution on Pancreatic Cancer Cells In Vitro and In Vivo. Ann. Surg. Oncol..

[30] Iseki S., Nakamura K., Hayashi M., Tanaka H., Kondo H., Kajiyama H., Kano H., Kikkawa F., Hori M. (2012). Selective killing of ovarian cancer cells through induction of apoptosis by nonequilibrium atmospheric pressure plasma. Appl. Phys. Lett..

[31] Kajiyama H., Utsumi F., Nakamura K., Tanaka H., Mizuno M., Toyokuni S., Hori M., Kikkawa F. (2016). Possible therapeutic option of aqueous plasma for refractory ovarian cancer. Clin. Plasma Med..

[32] Dong B., Bauchire J.M., Pouvesle J.M., Magnier P., Hong D. (2008). Experimental study of a DBD surface discharge for the active control of subsonic airflow. J. Phys. D Appl. Phys..

[33] DeBerardinis R.J., Chandel N.S. (2016). Fundamentals of cancer metabolism. Sci. Adv..

[34] Kurake N., Tanaka H., Ishikawa K., Kondo T., Sekine M., Nakamura K., Kajiyama H., Kikkawa F., Mizuno M., Hori M. (2016). Cell survival of glioblastoma grown in medium containing hydrogen peroxide and/or nitrite, or in plasma-activated medium. Arch. Biochem. Biophys..

[35] Canal C., Fontelo R., Hamouda I., Guillem-Marti J., Cvelbar U., Ginebra M.P. (2017). Plasma-induced selectivity in bone cancer cells death. Free Radic. Biol. Med..

[36] Reuter S., Von Woedtke T., Weltmann K.D. (2018). The kINPen—A review on physics and chemistry of the atmospheric pressure plasma jet and its applications. J. Phys. D Appl. Phys..

[37] Lu P., Boehm D., Bourke P., Cullen P.J. (2017). Achieving reactive species specificity within plasma-activated water through selective generation using air spark and glow discharges. Plasma Process. Polym..

[38] Machala Z., Tarabová B., Sersenová D., Janda M., Hensel K. (2019). Chemical and antibacterial effects of plasma activated water: Correlation with gaseous and aqueous reactive oxygen and nitrogen species, plasma sources and air flow conditions. J. Phys. D Appl. Phys..

[39] Simek M., De Benedictis S., Dilecce G., Babický V., Clupek M., Sunka P. (2002). Time and space resolved analysis of N_2_(C^3^Π_u_) vibrational distributions in pulsed positive corona discharge. J. Phys. D Appl. Phys..

[40] Trachootham D., Alexandre J., Huang P. (2009). Targeting cancer cells by ROS-mediated mechanisms: A radical therapeutic approach?. Nat. Rev. Drug Discov..

[41] Takeda S., Yamada S., Hattori N., Nakamura K., Tanaka H., Kajiyama H., Kanda M., Kobayashi D., Tanaka C., Fujii T. (2017). Intraperitoneal Administration of Plasma-Activated Medium: Proposal of a Novel Treatment Option for Peritoneal Metastasis From Gastric Cancer. Ann. Surg. Oncol..

[42] Yeung T.L., Leung C.S., Yip K.P., Au Yeung C.L., Wong S.T.C., Mok S.C. (2015). Cellular and molecular processes in ovarian cancer metastasis. A Review in the Theme: Cell and Molecular Processes in Cancer Metastasis. Am. J. Physiol. Physiol..

[43] Crestale L., Laurita R., Liguori A., Stancampiano A., Talmon M., Bisag A., Gherardi M., Amoruso A., Colombo V., Fresu L. (2018). Cold Atmospheric Pressure Plasma Treatment Modulates Human Monocytes/Macrophages Responsiveness. Plasma.

